# Dental Unit Waterlines in Quito and Caracas Contaminated with Nontuberculous Mycobacteria: A Potential Health Risk in Dental Practice

**DOI:** 10.3390/ijerph17072348

**Published:** 2020-03-31

**Authors:** Orlando J. Castellano Realpe, Johanna C. Gutiérrez, Deisy A. Sierra, Lourdes A. Pazmiño Martínez, Yrneh Y. Prado Palacios, Gustavo Echeverría, Jacobus H. de Waard

**Affiliations:** 1 Facultad de Ciencias Químicas, Universidad Central del Ecuador, 170521 Quito, Ecuador; 2 Facultad de Medicina. Escuela de Bioanálisis, Universidad Central de Venezuela, 1053 Caracas, Venezuela; 3 Instituto de Biomedicina “Dr. Jacinto Convit”, Universidad Central de Venezuela, 1010 Caracas, Venezuela; 4 Instituto de Investigación en Salud Pública y Zoonosis CIZ, Universidad Central del Ecuador, 170521 Quito, Ecuador; 5 One Health Research Group, Facultad de Ciencias de la Salud, Universidad De Las Américas, 170504 Quito, Ecuador

**Keywords:** dental unit waterline (DUWL), biofilm, dental infection control, heterotrophic plate count (HPC), coliform bacteria, *Pseudomonas aeruginosa*, nontuberculous mycobacteria (NTM)

## Abstract

Three cases of severe odontogenic infections due to nontuberculous mycobacteria (NTM) in Venezuela that were directly associated with dental procedures and the finding of dental unit waterlines (DUWLs) in dental offices that were colonized with mycobacteria species was the reason for assessing the water quality of DUWLs in dental offices in two capital cities in South America, namely, Quito and Caracas. The main water supplies and the water from 143 DUWLs in both cities were sampled and especially checked for contamination with NTM. To measure the overall bacteriological quality of the water also the presence of heterotrophic bacteria, coliform bacteria, and *Pseudomonas* was determined. Results showed that respectively 3% and 56% of the DUWLs in Quito and Caracas yielded NTM species (up to 1000 colony-forming units (CFU)/mL). Furthermore, high and unacceptable total viable counts of heterotrophic bacteria and/or coliform bacteria and *Pseudomonas* were detected in 73% of the samples. We conclude that, in both cities, the water in the majority of DUWLs was contaminated with NTM and other potential pathogens, presenting a risk to human health. The detection of NTM in DUWL water with acceptable heterotrophic bacteria counts shows the need to include NTM in water quality testing. Mycobacteria are more resistant to disinfection procedures than other types of vegetative bacteria, and most testing protocols for DUWLs do not assess mycobacteria and thus do not guarantee risk-free water.

## 1. Introduction

Dental unit waterlines (DUWL) provide water for irrigation, cooling, and flushing of the patient’s oral cavity during dental procedures. Because patients and dental staff are regularly exposed to this water and the aerosols generated from the dental unit, the microbial quality of this water is of importance because contamination with pathogens can pose a health risk, especially for elderly and immunocompromised people [1]. The most discussed infection event due to DUWL water is a fatal case in 2012 of a healthy woman who developed Legionnaire’s disease after a dental visit [2]. Since then, the presence of *Legionella* spp. in DUWLs has been reported in several studies in Europe, showing prevalence rates up to 53% [3,4]. The narrow waterlines of DUWLs provide a favorable environment for biofilm formation, and the water in these systems is therefore often contaminated with high densities of bacteria, fungi, protozoa, and viruses [1]. This has been confirmed by dozens of published articles during the last 50 years, and has been reviewed to some extent [5]. Furthermore, cross-infections with pathogenic microorganisms found in DUWLs have been reported in dentistry, such as the herpes simplex, varicella-zoster, hepatitis virus, *Pseudomonas* spp., and multiresistant bacteria [6].

Awareness of the infection risk due to DUWLs has led the Centers for Disease Control and Prevention (CDC) and the American Dental Association (ADA) to address the topic of water quality in their infection control guidelines [7,8,9,10]. The present recommendation in the USA for dental unit water used in nonsurgical procedures is that this water should measure less than 500 colony-forming units (CFU) of heterotrophic microorganisms per milliliter (heterotrophic plate count (HPC) ≤ 500 CFU/mL). This is also the standard for drinking water set by the Environmental Protection Agency (EPA) in the USA [11]. These guidelines also stipulate that sterile water should be used when performing surgical procedures [7,8,9]. To fulfill drinking water standards, the water in DUWLs should also be free of potential bacterial pathogens like coliforms. However, for many other potential bacterial pathogens, such as *Legionella* spp., *Pseudomonas*, and *Mycobacterium* species, no special recommendations can be found in most of the existing water quality guidelines as these are not considered bacteriological indicators of contamination and in general are not subject to drinking water regulations.

Concerning *Mycobacterium* species, these are opportunistic pathogens that are found ubiquitously in water and soil, and their presence in water samples collected from DUWLs has been reported [12,13]. There is evidence that waterlines colonized with these bacteria may bring about the risk of infection. Recently, in the USA, two outbreaks of odontogenic infections due to *Mycobacterium* species were reported. In Georgia, in 2015, 24 children were diagnosed with a severe infection due to *Mycobacterium abscessus* after a pulpotomy procedure carried out in a pediatric dentistry clinic, and 17 children required antibiotic treatment and the surgical excision of infected tissue. Examination of the water used during the procedure showed bacterial contamination far in excess of allowable concentrations, and *M. abscessus* was isolated from all water samples of the main supplies and DUWLs [14]. In 2016, the largest outbreak of odontogenic mycobacterial infections described to date was registered in California due to a pediatric dental clinic’s contaminated water system. Abnormalities of the mandible or maxilla, pain, swelling, lymphadenopathy, and pulmonary nodules caused by infection with *M. abscessus* were reported and affected at least 71 children [15]. The same species of NTM was also found in the DUWLs of the dental clinic where the children were treated. Moreover, in Caracas, Venezuela, three cases of severe infections with *M. fortuitum*, *M. peregrinum*, and *M. abscessus* were diagnosed in adult patients, with the infections directly associated with dental procedures and DUWLs contaminated with *Mycobacterium* species [16].

The aim of the present study was to assess the water quality of DUWLs in Quito, Ecuador, and Caracas, Venezuela, two capital cities in South America. We determined the general bacteriological quality of the water in the DUWLs and looked for specific pathogens, namely, thermoresistant coliforms and *Pseudomonas*, as well as the presence of Nontuberculous Mycobacteria (NTM) species, with the latter being the main variable of interest in this study.

## 2. Materials and Methods

### 2.1. Study Design

From May to September 2018, the DUWLs of 143 dental chairs were sampled: 100 DUWLs of individual dentist offices in Quito and 43 DUWLs of 14 dental centers in Caracas. All offices or centers practiced only common dental practices like bridges, implants, crowns, fillings, repairs, and extractions. No oral or maxillofacial surgery was practiced in any of the dental offices or centers that were visited. The dental offices in Quito were distributed over most (22) of the 32 urban parishes of this city and were chosen randomly from a list of available dental offices. The dental health centers in Caracas were located in 4 of the 10 urban districts of the city and were a convenient sample of dental centers that were easily reachable by public transport and willing to collaborate with this investigation. In both cities, the dental operative units were sourced with chlorinated drinking water from the municipal water supplies.

### 2.2. Sampling of the Waterlines from the Dental Unit and the Mains

Sampling was performed during working hours when the dentist had already seen a number of patients and the water from the syringe had been allowed to run for at least several minutes. Before sampling, the outside of the nozzle tip of the dental triple syringe and the water tap of the mains were wiped with a cotton gauze drenched in 70% ethanol, and the waterlines were then flushed for 30 s to aid in physically flushing out patient material that may have entered the waterlines [17]. Approximately 1 L of water was subsequently sampled from the main water supply and the DUWL. Samples were collected in previously autoclaved containers, transferred to the laboratory in insulated boxes with cooling packs, and stored at 4 °C until processing. Residual chlorine in the water samples was not inactivated with sodium thiosulfate as is usually done when analyzing water samples because this apparently affects the viability of some *Mycobacterium* species [18], and the isolation of this microorganism was the main objective of this research. In general, the samples were processed within a maximum period of 48 h. The dental offices participated voluntarily in this study and were informed about the results of this investigation. The research teams of both cities were calibrated and followed the same protocol.

### 2.3. Bacteriological Analysis of the Water Samples

Microbiological analysis of the water samples in Quito, Ecuador, was conducted in the laboratories of the “Instituto de Investigación en Salud Pública y Zoonosis”. The samples from Caracas were processed in the “Laboratorio de Tuberculosis” of the “Instituto de Biomedicina”. Care was taken to avoid cross-contamination, and trained students and professionals handled the samples aseptically.

For the heterotrophic plate count, colony-forming unit counts of heterotrophic microorganisms were determined with the spread plate technique on Reasoner’s 2A (R2A) agar and 100 µL inoculation of water as described by the American Public Health Association (APHA) [19]. Colonies were counted after incubation at 37 °C for 3 days.

For the detection of specific pathogens, coliforms, mycobacteria, and *Pseudomonas* were isolated by processing 200 mL of water using the membrane filtration technique with a 0.45 µm nitrocellulose filter as described by the APHA [19]. For the detection of total coliforms and thermotolerant coliforms or *Escherichia coli*, the filters were placed on MacConkey agar (Venezuela) or Endo agar plates (Ecuador) and incubated at 37 °C for 24 h. Isolates were further identified as thermoresistant (fermentation of lactose at 42 °C) and identified at a species level as *E. coli* with standard microbiology techniques. Detection of *Pseudomonas* was performed only in Caracas. A volume of 200 mL of water was filtered, and the filters were placed on selective Cetrimide agar medium and incubated for 48 h at 37 °C. Isolates producing blue-green and yellow-green pigments, visible under ultraviolet light (254 nm), were identified at a species level as *Pseudomonas aeruginosa* with standard microbiology techniques. For the isolation of mycobacteria, 200 mL of water was decontaminated to eliminate Gram-positive/negative bacteria and molds with hexadecylpyridinium chloride at a final concentration of 0.17% for 20 min [20]. The decontaminated water was then filtered through a 0.45 µm nitrocellulose filter, and a further 100 mL of sterile water was passed through the filter to neutralize it for the HPC detergent. This filter was then incubated, upside down, for two days in a Petri dish with TSA (Trypticase soy broth with agar) supplemented with 0.5% of glycerol and a mixture of antibiotics VAN (vancomycin, amphotericin B and nalidixic acid) in Venezuela and PANTA (polymyxin B, amphotericin B, nalidixic acid, trimethoprim and azlocillin) in Ecuador). For the concentration of both antibiotic mixtures, see [21]. This antibiotic mixture was used to overcome the growth of any molds or bacteria that were not mycobacteria and were not killed by the HPC decontamination procedure. After two days, the filter was removed, and the Petri disks were incubated for another 4 weeks until the growth of bacteria was observed. Mycobacteria were identified with the acid-fast staining technique and to a species level with the PCR-restriction enzyme analysis (PRA)-hsp65 method following the protocol established by Telenti et al. [22].

### 2.4. Survey on Disinfection Practices

The dentists were interviewed concerning disinfection practices and quality control of the DUWL water. The following four close-ended questions had to be answered. Do you monitor the bacteriological water quality of the DUWL? Did you disinfect the DUWL recently? How often do you disinfect the waterlines? Which product do you use to maintain the waterlines free of biofilm formation? The latter two questions were only asked where applicable.

## 3. Results

### 3.1. Water Quality in Quito

In Quito, 100 dental offices, all with one single chair, were evaluated, and 31 (31%) DUWL samples had a HPC lower than 500 CFU/mL. Sixty-nine (69%) of the DUWLs exceeded this guideline value with an average count of 8350 CFU/mL. Twenty-four chairs had counts of up to 5000 CFU/mL, 33 DUWLs had counts between 5000 and 15,000 CFU/mL, and 12 had counts higher than 15,000 CFU/mL. Of the samples, 14% yielded coliforms identified as *E. coli* (Table 1). The presence of this regulated drinking water contaminant provides evidence of fecal contamination. Three chairs (3%) presented contamination (10–500 CFU/200 mL) with species of NTM, identified by the PRA-hsp65 method as *M. abscessus* (two isolates) and *M. fortuitum* (one isolate). Concerning the source water for the chairs, in Quito, 40% of the chairs were connected to the municipal water system, and the others had a closed system with independent water tanks, which were filled with municipal water or with drinking water of commercial brands. All the main supplies or commercial drinking water used to fill the reservoirs of the chairs had a low number of HPC microorganisms (maximum 25 CFU/mL), and no NTM or other pathogens were isolated from these samples (Table 1).

### 3.2. Water Quality in Caracas

**Figure 1:**
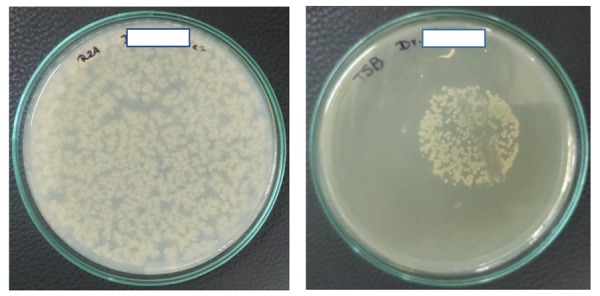
Examples of positive water cultures. On the left, 100 µL of water was plated on Reasoner’s 2A (R2A) agar, and the Petri dish was incubated at 36–37 °C for 72 h. On the right is the result of a water sample that was positive for mycobacteria after 7 days of growth at 37 °C. An uncountable number of mycobacteria was isolated from 200 mL of DUWL water. The mycobacteria were isolated using the membrane filtration technique with a 0.45 µm nitrocellulose filter with a diameter of 47 mm, that was placed upside down on TSB agar with glycerol and an antibiotic mix, and again removed after two days of preincubation (see Materials and Methods). The visible streak through the colonies on this plate is where a loop full of bacteria had been taken for confirmation of the presence of acid-fast bacilli using the Ziehl–Neelsen staining technique. The mycobacteria on this plate were identified with the PCR-restriction enzyme analysis (PRA) technique as *M. abscessus*. For the purpose of anonymity, the name of the dentist on the Petri dish has been hidden.

In Caracas, 14 dental centers (with a total of 43 chairs) were evaluated. All chairs had a closed tank system that was filled with water from the municipal water system or commercially available mineral water (three chairs of one dental center). Analysis of the water demonstrated the presence of aerobic mesophilic heterotrophic bacteria in the main supplies and in triple syringes, with HPC ranging from 200 to 16,000 CFU/mL, and 64% of the mains and 84% of the DUWLs exceeded the established international standards of 500 CFU/mL (Table 1). A total of 21% of the mains and 23% of the DUWLs yielded *E. coli*, which is evidence of fecal contamination. We also cultivated for *Pseudomonas* in Caracas, and the presence of *Pseudomonas aeruginosa* was demonstrated in 29% and 51% of the water samples of the main supplies and the DUWLs, respectively, with counts between 17 and 1800 CFU/ 200 mL. In five of the 14 centers, between 58 and 120,000 CFU/200 mL of NTM were isolated from water of the main supplies. The DUWLs of 24 of the 43 chairs yielded mycobacteria with colony counts between 1 and 190,000 CFU/200 mL. *M. fortuitum* was the only NTM species isolated from the main supplies. The DUWLs yielded mycobacteria species that were identified as *M. fortuitum* (44%), *M. chelonae* (28%), *M. abscessus* (16%), *M. brisbanense* (4%), *M. peregrinum* type 3 (4%), and *Mycobacterium* spp. (4% of the isolates). Two DUWLs presented the growth of two species of NTM. An example of a HPC Petri dish and a Petri dish with isolated mycobacteria, both from Quito water samples, is given in Figure 1.

### 3.3. Comparison of Water Quality in DUWLs in Caracas and Quito

The bacteriological results for both cities are summarized in Table 1, which permits a comparison of the results between the two cities. It is clear from this table that, in Caracas, there was a direct link between water quality from the main water supply and water quality from the DUWLs. In Quito, certain bacteria, not present in the dental offices’ main water supplies (coliforms and NTM species), were found in the water of the DUWLs, indicating biofilm formation in the waterlines of these devices.

### 3.4. Disinfection Practices of DUWLs in Quito and Caracas

In both cities, none of the DUWLs had ever been tested for the presence of microorganisms. In addition, the majority of the dentists (76%) were not aware of the possibility of testing water quality. Only one dental center with five chairs in Caracas and 30 dentists (30%) in Quito stated that they disinfect the waterlines once every 1–2 months with sodium hypochlorite (concentrations between 0.1% and 2%) or chlorhexidine gluconate (used by 4% of the dentists in Quito). In general, HPC in DUWLs that were disinfected were lower, and the only dentist in Caracas that followed the recommended decontamination procedures and disinfected with 0.5% sodium hypochlorite had acceptable HPC. However, although water used to source these DUWLs did not yield NTM, *M. fortuitum* was isolated from all the five DUWLs of this office in Caracas. In addition, all the chairs in Quito that followed a disinfection procedure (30/100) had acceptable HPC. However, NTM species were isolated from the water of the triple syringe from the chairs of three of the four dentists who used chlorhexidine gluconate in decontamination procedures, demonstrating that NTM are resistant to this disinfectant. No coliforms were isolated from chairs where waterlines were disinfected.

## 4. Discussion

This study examined the microbial quality of water from DUWLs in two cities, namely, Quito and Caracas. Results showed that 73% of the DUWLs had a high microbial load of aerobic mesophilic heterotrophic bacteria. Concerning pathogenic bacteria, NTM were isolated in 19% of the DUWL samples, and we also found considerable loads of *P. aeruginosa* and coliform bacteria, especially the fecal bacteria *E. coli*.

### 4.1. General Microbiological Quality of Water in the DUWLs

We included testing of the sources of water supplies to the chairs, which was in most cases chlorinated drinking water and came from the water distribution systems of Quito and Caracas. The drinking water in Quito was of good quality, complied with bacterial norms for drinking water, and was thus suitable for use in the DUWLs; no NTM were isolated. In Caracas, the water sources were mostly household water tanks because of the difficulties of a constant and reliable municipal water supply in this city. Water from these sources was found to be heavily contaminated and should not be used as water for DUWLs. Commercially available mineral water or sterilized water should be used for the sealed water tank systems that were present in all the DUWLs we investigated in this city. Only four dental chairs in Caracas used mineral water that was not analyzed in this study. However, no significant difference in contamination rates was found for water of the DUWLs that used mineral water.

We observed that, in both cities, there was a greater degree of contamination in the water of the DUWLs than in the water of the main supplies (Table 1). This may be due to the presence of biofilms of microorganisms in the units as the narrow dental unit waterlines are an ideal environment for the development of microbial biofilms [1,10]. The ADA and CDC therefore recommend regular disinfection of DUWLs, but most dentists in our study did not disinfect their DUWLs and were not aware of the possibility of testing. This shows the need for educational programs and the installation of governmental control mechanisms.

Very few studies in Latin America have assessed water quality in dental offices. In fact, after searching the PubMed database, we found only three publications in the English language, all from Brazil [23,24,25]. In a 2003 study, 14 of the 15 dental units from private dental clinics in São Paolo had HPC measurements of approximately 50,000 to 300,000,000 microorganisms per milliliter [23]. In 2008, contamination of the waterlines with *Pseudomonas* was reported in 20 dental clinics in São Paulo, and 28 out of 40 samples of water syringes had HPC measurements between 200 and 2000 CFU/mL [24]. More recently, in 2017, another study from Brazil reported fungal contamination of DUWLs [25]. The presence of mycobacteria was not assessed in these studies from Brazil. Meanwhile, in Europe, there have been many more studies concerning the problem of contaminated water in DUWLs. In Germany, for instance, a study analyzed the microbial quality of water from 56 dental units. Contamination by *Legionella* species and *Pseudomonas aeruginosa* and increased total colony counts were detected in 27.8%, 3.5%, and 17% of DUWL samples, respectively [26]. A study in Italy in 2018 compared DUWLs directly connected to the municipal water supply with chairs with a self-contained water system and found *Pseudomonas* in 25.0% and 77.4% of the tested DUWLs respectively. Legionella was isolated in 37.5% and 91.7% of the DUWLs, respectively [27]. In this Italian study, the presence of microorganisms was linked to water supply characteristics, and the source water provided from a water tank resulted in more heavily contaminated DUWLs than those directly connected to municipal water. Water tank systems provide a means for introducing chemical agents to waterlines and permit the use of water of known microbiological quality [28]. However, in this study in Italy, without chemical treatment to remove or inactivate the biofilm within the water tank, a self-contained water system reduced the microbial quality of dental unit water and DUWLs using municipal water in this study were less prone to contamination. The extended retention time and stagnation of the water in DUWLs with a self-contained water system will increase biofilm activity. These findings are in agreement with our study, where the direct use of municipal water in Quito resulted in less contamination than the use of municipal water stored in household water tanks in Caracas.

### 4.2. Nontuberculous Mycobacteria in DUWLs

Regarding *Mycobacterium* species in DUWLs, the presence of these microorganisms is well documented in drinking water samples, but they are in general not considered bacteriological indicators of contamination for drinking water or DUWLs. Our research shows that they can be isolated and identified from water in DUWLs. Moreover, outbreaks due to these bacteria after odontological care have recently been reported I the USA and in Venezuela [14,15,16].

Only a few studies in the scientific literature have previously investigated the presence of mycobacteria in DUWLs [12,13,29]. In the United Kingdom, where 95% of DUWL water samples exceeded European Union drinking water guidelines for microbial load, *Mycobacterium* species were isolated from 10% of the water samples. These isolates were not identified, so their pathogenic potential is unknown [29]. In the United States, in a hospital dentistry clinic in Texas, two species of mycobacteria, namely, *M. simiae* and *M. mucogenicum*, were detected in the DUWL before and after treatment with an iodine-containing waterline cleaner [13]. A study in Germany found all of the 43 water samples of DUWLs were positive for mycobacteria, and the mean mycobacteria concentration was 365 CFU/mL [12] in DUWLs that were being treated daily with a disinfectant containing a low concentration of hydrogen peroxide (0.93%) or chlorine. These studies in the USA and Germany showed the failure of the disinfection procedure for the DUWLs regarding mycobacteria. Mycobacteria are more resistant to disinfection than viruses and other types of vegetative bacteria, and only high-level disinfectants in an adequate concentration can kill mycobacteria. Iodophores (used in the USA study), or a low concentration of hydrogen peroxide (used in the study in Germany) have a moderate activity against mycobacteria, and prolonged incubation is needed for full activity. Iodophores are intermediate-level disinfectants and hydrogen peroxide should be used at a concentration of 7% or more to acquire high-level disinfection [30]. Moreover, the use of an intermediate-level disinfectant can select for a more resistant NTM phenotype that appears at high frequency. This has been shown for disinfection with quaternary ammonium compounds, which selects for a quaternary ammonium compound (QAC)-resistant NTM phenotype [31]. In our study, in Quito and Caracas, the use of an intermediate-level disinfectant, namely, chlorhexidine gluconate or low concentrations of sodium hypochlorite, for the decontamination of DUWLs resulted in acceptable HPC. However mycobacteria were isolated from the DUWLs that were not present in the source water.

All studies looking at mycobacteria in the DUWLs show that high numbers of mycobacteria may be swallowed, inhaled, or inoculated into oral wounds during dental treatment, possibly resulting in colonization, sensitization, or infection [13]. Concerning an adequate disinfectant for DUWLs, especially to eliminate mycobacteria, no recommendations can be found in the existing guidelines. Moreover, disinfection guidelines for DUWLs, as far as we know, do not include the elimination of mycobacteria, and NTM are generally not included as a parameter in standard microbiological analysis. This is in strong contrast to the disinfection procedures in medical practice, where disinfection of semicritical instruments should be performed with a high-level disinfectant, defined as a disinfectant with a tuberculocidal activity, which eliminates mycobacteria [32,33].

As for the risk of infection, we are aware that it is difficult to estimate the potential risk derived from microorganisms isolated from DUWLs, and the risk of transmitting pathogens in a dental office is difficult to assess, although it cannot be considered negligible [34,35]. Furthermore, exposing patients or the dental team to contaminated water is not consistent with universally accepted infection-control principles [34].

Concerning the minimum infective dose needed to establish an infection with NTM, no information is available, but most mycobacteria are considered highly infectious. For members of the *Mycobacterium tuberculosis* complex strains, it was found that one CFU of *M. bovis* was sufficient to establish tuberculous pathology in cattle [36]. Nearly 30% of mice exposed to a 1 CFU of *M. tuberculosis* got infected [37]. For NTM, no specific infective dose has been determined yet, and virulence will most probably differ between mycobacterial species. Yet, for *M. abscessus*, an NTM species isolated from the DUWLs in Quito and Caracas, less than 10 mycobacteria were sufficient to establish soft tissue infections in humans practicing mesotherapy and administrated subcutaneous injections of 0.1 mL with a solution contaminated with 100 CFU/mL of NTM (personal communication JHdW and [38]).

## 5. Conclusions

The high microbial loads of aerobic mesophilic heterotrophic bacteria as well as the pathogenic *P. aeruginosa*, coliform bacteria and NTM in DUWLs of most dentists show that, in both Quito and Caracas, there is a need to disinfect DUWLs and to regularly monitor the microbial quality of the water. The presence of NTM in the water should also be monitored after disinfection of the waterlines as the use of a low-level disinfectant will remove most bacteria but can lead to an accumulation of NTM. In Caracas, Venezuela, and also in the USA, outbreaks of NTM infections have been registered as being associated with NTM-contaminated water in DUWLs [14,15,16]. In order to keep patients safe, one must use water of an appropriate microbiological quality or sterile water, especially when surgical procedures are performed and/or when treating certain groups of people, e.g., immunocompromised and older patients. Our results are a warning signal and a call for the application of biosecurity measures. Infection control in dental offices should be considered an essential issue, and dentists should understand the limitations of available DUWL treatments.

## 6. Limitations of This Study

In microbiological water quality testing, sample dechlorination with sodium thiosulfate is recommended to ensure that results accurately reflect the water quality. In general, samples held without sodium thiosulfate have lower bacterial counts. Our samples were collected without sodium thiosulfate to support the isolation of mycobacteria [18]. Therefore, the results of the viability count of our water samples for other bacteria would most probably have been higher if the samples had been dechlorinated [39]. This also applies to the holding time of our water samples. For logistical reasons, about 30% of the samples were processed outside of the time limit stipulated by international guidelines (24 h). When the holding time of the water before processing exceeds this time limit, the viability of most of the bacteria present in the water will diminish, and an underestimation of the real contamination level will consequently occur [40,41]. Moreover, our culturing techniques do not exclude the presence of other pathogenic bacteria in DUWLs, such as *Legionella* spp. or what are currently the most problematic healthcare-associated multiresistant species, i.e., methicillin-resistant *Staphylococcus aureus* (MRSA), extended-spectrum beta-lactamases (ESBL)-producing *Enterobacteriaceae,* and other carbapenemase-producing Gram-negative bacteria. Antimicrobial susceptibility testing of the *E. coli* and *P. aeruginosa* strains isolated in this study has not been realized as we considered this to be outside the scope of this article. Furthermore, while *E. coli* is a useful indicator of fecal contamination, we did not look for enteric viruses or for protozoa, and bacterial spores, which are in general more resistant to disinfection and are possibly present in the waterlines.

## Figures and Tables

**Table 1:** Presence of bacteria in the mains and dental unit waterlines (DUWLs) in Quito and Caracas.

	HPC >500 CFU/mL Main/DUWL		Presence of Coliforms ** % Main/DUWL		Presence of *P. aeruginosa* % Main/DUWL		Presence of Mycobacteria % Main/DUWL	
Quito	0%	69%	0%	14%	ND *	ND *	0%	3%
Caracas	64%	84%	21%	23%	29%	51%	36%	56%

Note: Microbiological results of 100 dental offices in Quito and 14 dental centers in Caracas. In Quito, 100 mains and 100 DUWLs were sampled. In Caracas, the results consist of 14 mains supplying the respective dental centers and 43 DUWLs. * ND = not determined. ** In Quito and Caracas, all samples that gave positive results for coliforms also yielded fecal coliforms (lactose fermentation at 42 °C) and *E. coli* as determined by standard microbiological techniques in Caracas or “colonies producing a green metallic sheen on Endo agar” that was used for identification in Quito.

## Abbreviations

DUWL	dental unit waterline
HPC	heterotrophic plate count
CFU/mL	colony-forming units per milliliter
NTM	nontuberculous mycobacteria
PRA technique	PCR-restriction enzyme analysis
ADA	American Dental Association
CDC	Centers for Disease Control and Prevention
